# Improved Method for Quantitative Measurement of OH Radicals Based on Absorption Spectroscopy

**DOI:** 10.3390/molecules31010118

**Published:** 2025-12-29

**Authors:** Xiu Yang, Jie Cui, Rui Ma, Lindan Yue, Yongzhuo Yin, Janhua Qi, Youning Xu, Benchuan Xu, Liang Zhu

**Affiliations:** 1 Key Laboratory of Liaoning Province for Clean Combustion Power Generation and Heating Supply Technology, Shenyang Institute of Engineering, Shenyang 110136, China; 12023101116@stu.sie.edu.cn (X.Y.);; 2 Institute of Power Plant Technology, Steam and Gas Turbines, RWTH Aachen University, 52074 Aachen, Germany; 3 Shenyang Qingjie Energy Technology Co., Ltd., Shenyang 110136, China; 4 Liaoyang Guocheng Thermal Power Co., Ltd., Liaoyang 111003, China; 5 Xinjiang Saier Shan Municipal Engineering Co., Ltd., Tacheng 839099, China

**Keywords:** OH radical, OH-PLIF, absorption spectroscopy, two-line temperature inversion

## Abstract

OH-PLIF quantitative measurements suffer from high temperature sensitivity and poor applicability of calibration constants, this paper combines absorption spectroscopy with dual-line temperature inversion to establish an explicitly temperature-corrected OH radical concentration inversion model. By simultaneously acquiring PLIF images and absorption spectrum data under varying hydrogen-oxygen mixture flow rates, the equivalent absorption path length is calculated and the temperature-dependent absorption cross-section σ(*ν*,*T*) is incorporated. This enables the dynamic response of the integral absorption rate to high-temperature flame environments. Results demonstrate that the established temperature-corrected model significantly reduces systematic errors caused by temperature variations, with calibration constant *C* fluctuating less than ±5% across different operating conditions. Further optimization via least-squares method yielded the optimal constant *C_opt_* = 0.01844. Its applicability was validated across various operating conditions, with average relative errors controlled within 4–6%. Compared to the uncorrected model, overall error decreased from 9.1% to 5.2%.

## 1. Introduction

The hydroxyl (OH) radical is a key intermediate species in combustion reactions, and its spatial concentration distribution and temporal evolution are crucial for understanding flame stability, reaction pathways, chemical kinetics, pollutant formation mechanisms, and heat release efficiency [1,2]. Moreover, OH radicals can reflect the temperature distribution and mixing uniformity within flames, serving as a reliable indicator for identifying reaction zones [3]. Therefore, accurate measurement of OH radical concentration is of great importance for elucidating hydrogen–oxygen combustion mechanisms, validating kinetic models, and optimizing burner structure and operating conditions.

Planar Laser-Induced Fluorescence (PLIF), owing to its non-intrusive nature and high spatial-temporal resolution, has been widely employed for combustion visualization and two-dimensional measurements of radical concentrations, becoming a core diagnostic tool in modern combustion research [4,5,6]. Rocha et al. [7] applied PLIF to measure the distribution of NH and NO in NH_3_/CH_4_/air premixed laminar flames under varying equivalence ratios and pressures using a pressurized Bunsen-type configuration. Sun et al. [8] investigated the effects of a gliding arc (GA) discharge on the lean blow-off limit and NO_x_ emissions of NH_3_/CH_4_/air premixed swirling flames. By combining OH-PLIF, particle image velocimetry (PIV), and NH_2_ fluorescence, they provided detailed insights into the structure and emission characteristics of plasma-assisted flames. Allison et al. [9] utilized simultaneous CH_2_O/OH dual-component PLIF to investigate the preheat, reaction, and high-temperature product zones in a dual-mode scramjet combustor, revealing the structural evolution of low-temperature fuel decomposition and flame reaction zones. However, these studies mainly focused on the visualization of flame structures and reaction zones, and the measured signals primarily reflected the spatial distribution of OH radicals without enabling absolute quantitative measurement. Consequently, researchers have gradually shifted their attention from qualitative two-dimensional imaging to quantitative calibration and analysis of PLIF signals. Hanson et al. [10] established a two-level excitation kinetic model for OH radicals, deriving the theoretical relationship between fluorescence intensity and number density, and elucidated the effects of optical efficiency, excitation volume, and collisional quenching on quantitative measurements. Building on this, Seitzman et al. [11] developed a two-dimensional quantitative imaging approach, significantly extending the application scope of PLIF in combustion diagnostics. Li [12] combined absorption spectroscopy with PLIF to quantify OH concentrations in n-heptane laminar flames by calculating the integrated absorbance of characteristic spectral lines, demonstrating the reliability of this method under high-temperature flame conditions. Chen et al. [13] established a linear relationship between fluorescence intensity and mole fraction in methane–air flames under fixed laser frequency and ICCD parameters, defining a calibration constant *C*, which exhibited less than 3% fluctuation under steady operating conditions. In addition, Cheskis and Goldman [14] employed high-resolution spectroscopy in low-pressure flat flames to systematically investigate the excitation kinetics of OH (A^2^Σ^+^-X^2^Π) transitions. They found that nonlinear quenching and self-absorption effects at low pressures and high energy densities significantly reduce the quantum efficiency, and proposed corrections to the effective quenching coefficient and excitation power density to improve quantitative precision. Matynia et al. [15] conducted absolute OH concentration calibration under high-pressure conditions by coupling LIF, PLIF, and absorption spectroscopy. Jalbert [16] performed systematic OH-PLIF quantitative experiments in laminar flat flames and proposed a multi-parameter normalization strategy based on laser energy, gray-level integration, and background noise correction. The results showed that the measurement error was reduced from 8% to below 3%, significantly improving the reproducibility of cross-condition measurements. With the advancement of spectral databases and simulation tools, Luque [17] developed the LIFBASE program, enabling standardized simulation of energy-level populations and spectral characteristics, thereby providing reliable parameters for OH, CH, and NO radicals in PLIF calibration.

In summary, although the existing OH-PLIF quantitative measurement systems have been well developed, their accuracy remains limited under highly reactive combustion conditions. Most previous models rely on calibration constants obtained under fixed-temperature or single-point conditions and fail to adequately account for the influence of temperature variation and collisional quenching on fluorescence signals, leading to deviations in the linear relationship between fluorescence intensity and OH concentration under different operating conditions.

In this study, hydrogen-oxygen premixed flames were selected as the research object, and a temperature-constrained multi-point calibration method was proposed by combining absorption spectroscopy with a two-line temperature inversion approach. By synchronously acquiring OH-PLIF signals under different hydrogen-oxygen flow conditions, a temperature-corrected calibration model was established. The quenching effects were further corrected using the results of the two-line temperature inversion, which significantly improved the accuracy and consistency of OH concentration retrieval. This work provides an experimental basis for quantitative measurement of radicals and for investigating combustion characteristics in hydrogen-oxygen flame environments.

## 2. Results and Discussion

### 2.1. Equivalent Absorption Path Length

**Figure 1:**
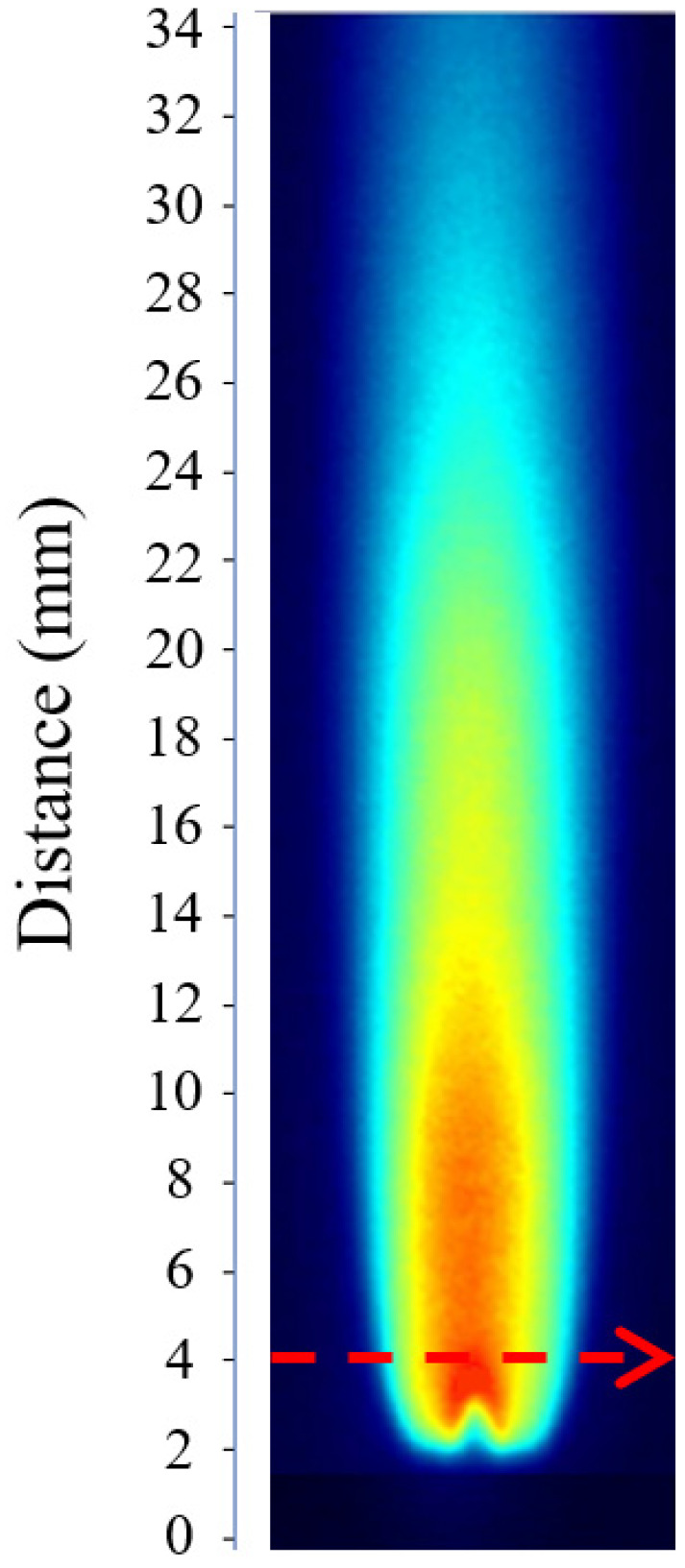
OH-PLIF image under calibration conditions used for determining the effective absorption path length *l*. (During the calibration process, the laser beam passes through the burner from left to right and is positioned 4 mm above the nozzle exit).

According to the analysis of Equation (3), determining the OH radical concentration requires the experimental measurement of the absorption path length *l*. The calculation of *l* relies on the PLIF image obtained under the calibration condition. Figure 1 shows the PLIF image corresponding to the calibration case, where the laser beam propagates from left to right across the burner at a height of 4 mm above the nozzle.

**Figure 2:**
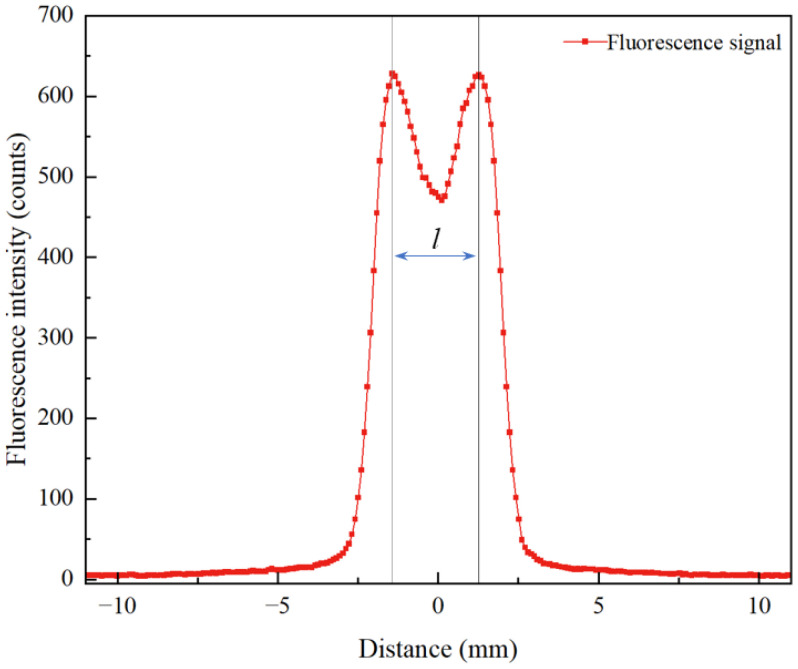
Fluorescence intensity distribution at 4 mm height.

As shown in Figure 2, the OH fluorescence intensity at the 4 mm height exhibits a bimodal distribution, requiring the calculation of an equivalent absorption path length. The *F*(*x*) data were imported into Python 2023 for numerical integration using the trapezoidal rule, yielding an integrated value of 1879.03 counts·mm. By taking the average OH fluorescence intensity at the 4 mm height as 580 counts, the equivalent absorption path length was determined to be *l* = 3.24 mm.

### 2.2. Integrated Absorption Rate and Temperature Inversion Results

To achieve the concentration inversion of OH radicals, this section conducts theoretical analysis and quantitative calculation based on the absorption experimental data of the Q_1_(8) transition line, using the Beer-Lambert law [18] and the integrated absorption rate model.

The dye laser output wavelength was locked at 283.62 nm, with an output laser energy of 20 mJ. The laser system was controlled via DaVis software, setting both the laser amplifier gain and ICCD camera gain to 75%, with a delay time of 700 ns and a gate width of 1000 ns. According to the laser power meter measurements, when the laser amplifier gain was 75%, the average incident laser energy on the hydrogen-oxygen flame was approximately 15 mJ, while the average transmitted laser energy was about 8.2 mJ, corresponding to an integrated absorption rate of *A* = 0.606.

**Figure 3:**
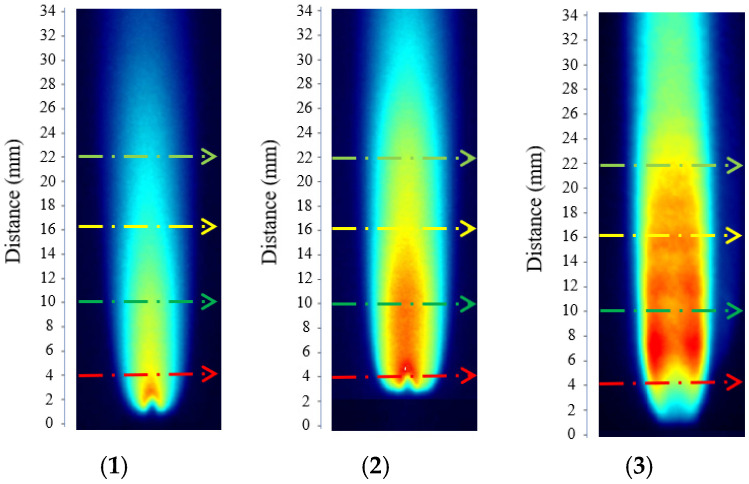
Annotated schematic diagram. ((**1**), (**2**), and (**3**) correspond to hydrogen-oxygen flow rates of 0.4 L/min, 0.8 L/min, and 1.2 L/min, respectively. In the figure, the red, green, yellow, and cyan dashed arrows represent flame exit heights of 4, 10, 16, and 22 mm, respectively.).

By performing temperature inversion at different operating conditions and flame heights, the results were obtained as listed in Table 1. Figure 3(1), (2), and (3) correspond to hydrogen-oxygen flow rates of 0.4 L/min, 0.8 L/min, and 1.2 L/min, respectively.

### 2.3. Calibration Results of Constant C and Applicability Analysis

The number density of OH radicals is determined by the integrated absorbance of the absorption line, excitation frequency, Einstein coefficient, and Boltzmann population fraction, among other parameters. To improve calculation accuracy, this study employed the Q_1_(8) transition parameters provided by the HITRAN database [19] in the computation process, combined with the experimentally obtained laser energy attenuation ratio from the PLIF measurements. The excitation wavelength was 283.62 nm, corresponding to an excitation frequency of approximately 1.058 × 10^15^ Hz. The Boltzmann population fraction was 0.0252, the Einstein coefficient was *A*_21_ = 1.45 × 10^6^ s^−1^, and Planck’s constant was h = 6.626 × 10^−34^ J/K.

To analyze the quantitative behavior under different flow conditions, three representative cases were selected for calculation, corresponding to hydrogen-oxygen mixture flow rates of 0.4 L/min, 0.8 L/min, and 1.2 L/min.

As summarized in Table 2, the calibration calculations were conducted under different hydrogen-oxygen mixture flow rates. For each operating condition, the incident and transmitted laser energies were measured, and the corresponding energy attenuation ratios, average OH fluorescence intensities at 4 mm, OH radical number densities, volume fractions, and calibration constants were determined. The tabulated results provide a clear and systematic comparison of the calibration parameters obtained at different flow rates.

**Figure 4:**
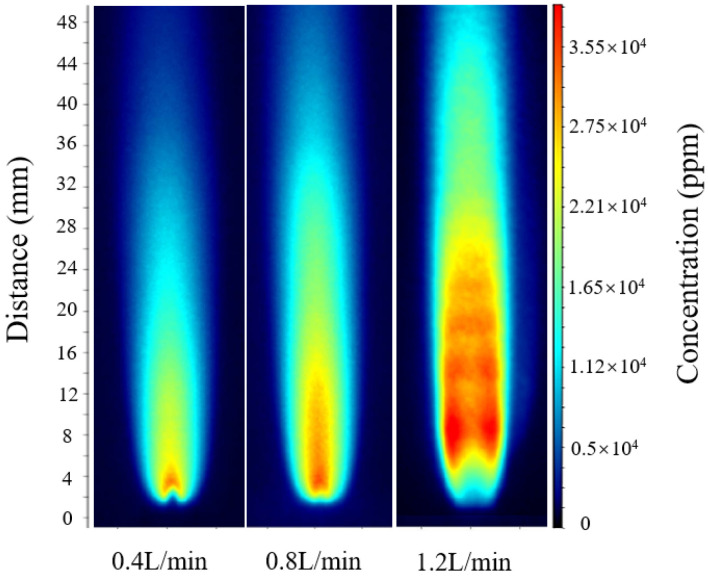
OH-PLIF images under hydrogen-oxygen mixed gas flow rates of 0.4, 0.8, and 1.2 L/min.

As shown in Figure 4, a comparison of the three operating conditions reveals that the laser energy attenuation ratio slightly increases with rising hydrogen-oxygen mixture flow rate, indicating that higher flow rates lead to elevated flame temperatures and higher OH radical concentrations, thereby enhancing absorption and reducing transmitted energy. However, the variation in the calibration constant *C* among the three conditions remains within approximately ±5%, demonstrating that the proposed temperature-correction model effectively compensates for absorption discrepancies caused by temperature gradients and local non-uniformities. As a result, the relationship between integrated absorbance and OH radical number density maintains good linear consistency across different operating conditions.

To ensure the applicability of the calibrated constant *C* under different operating conditions, the least-squares method was employed, yielding an optimal calibration constant of *C_opt_* = 0.01844. The least-squares analysis of the calibration data shows a strong linear agreement between the OH number density derived from absorption spectroscopy and the corresponding OH-PLIF intensity, with a coefficient of determination of *R*^2^ = 0.987.

**Figure 5:**
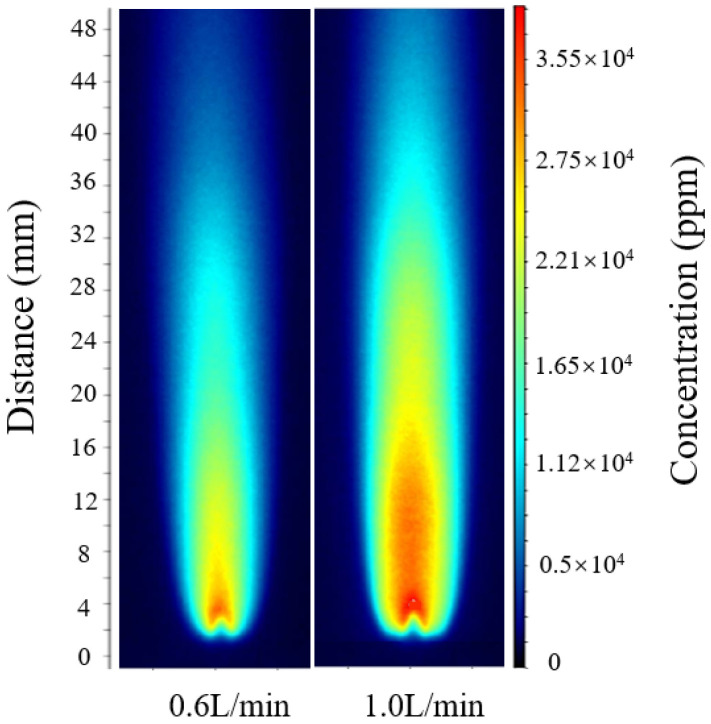
OH-PLIF images under hydrogen-oxygen mixed gas flow rates of 0.6 and 1.0 L/min.

According to the relative error calculation, the errors at flow rates of 0.4 L/min, 0.8 L/min, and 1.2 L/min were 4.9%, 5.6%, and 5.2%, respectively. The optimal calibration constant *C_opt_* was then applied to intermediate flow conditions that were not included in the fitting process for verification. As shown in Figure 5, for hydrogen-oxygen mixture flow rates of 0.6 L/min and 1.0 L/min, following the same calculation procedure, the maximum OH radical concentrations were approximately 30,690 ppm and 34,910 ppm, respectively.

The comparison results show that the inversion results for hydrogen-oxygen mixture flow rates of 0.6 L/min and 1.0 L/min both fall within the linear variation trend formed by the three operating conditions of 0.4, 0.8, and 1.2 L/min, with deviations from the calibrated trend values being less than 5%. This indicates that the optimal calibration constant *C_opt_* is equally applicable to other operating conditions. Further comparison of the full-field OH-PLIF distributions under different flow rates reveals that the peak position and attenuation profile of the OH radical concentration remain nearly identical, with the maximum relative deviation of about 5% and the average relative error controlled within 4–6%. These results confirm that the unified calibration model incorporating explicit temperature correction exhibits excellent adaptability and reliability across varying flow conditions.

A comparative calculation was conducted under identical conditions using the uncorrected form of the Matynia model (i.e., without considering the temperature dependence of σ(*ν*,*T*)). The results show that the average relative error of the uncorrected model is approximately 9.1%, whereas after introducing the temperature correction and applying the dual-line temperature inversion method, the error decreases to about 5.2%. This indicates that incorporating the temperature-dependent absorption cross-section effectively improves the linear correspondence between the integrated absorbance and OH number density, thereby enhancing the accuracy and applicability of the PLIF quantitative calibration.

## 3. Experimental System and Method

### 3.1. Experimental System

**Figure 6:**
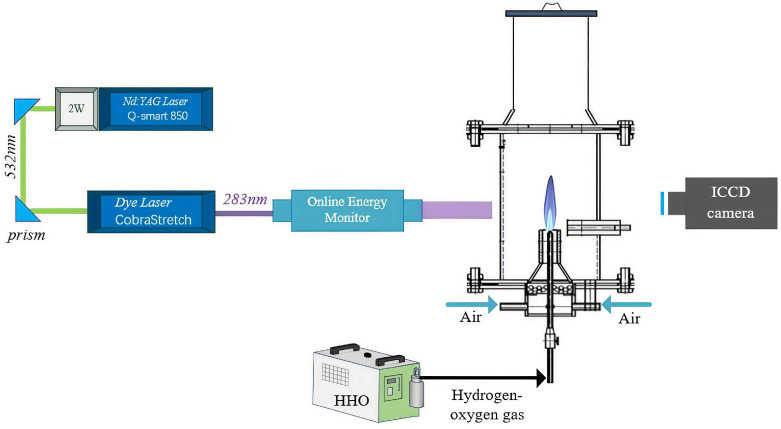
Experimental test platform.

The experimental system mainly consisted of a PLIF diagnostic setup, a burner, and a hydrogen-oxygen mixed gas generator, as shown in Figure 6. The PLIF diagnostic system comprised an Nd:YAG laser, a Sirah dye laser, optical lens assemblies, mirrors, an ICCD camera equipped with an optical filter, an online energy monitor, and the control software DaVis 10. After ignition of the hydrogen–oxygen premixed flame, the laser beam passed through a quartz tube and irradiated the flame. The excited OH radicals within the flame produced fluorescence, which was captured by the ICCD camera equipped with an OH band-pass filter, and the recorded images were post-processed using DaVis 10 software. The burner employed a variable-diameter nozzle with an outlet diameter of 5 mm. Because the hydrogen-oxygen premixed flame burns rapidly and is prone to flashback, a specially designed hydrogen-oxygen burner was adopted to ensure experimental safety. The burner mainly consisted of a nozzle, a needle valve, and a flame arrestor. The nozzle diameter was 0.8 mm, and a porous ceramic flame arrestor with pore diameters smaller than 0.5 mm (meeting the IIC safety standard) was installed between the valve and the outlet to prevent flashback and ensure stable combustion of the hydrogen-oxygen mixture. The hydrogen–oxygen gas was produced by a water-electrolysis generator (model 0H300) operating at 220 V, 50/60 Hz, with a rated power below 1.2 kW. The system generated approximately 300 L/h of gas, corresponding to an average flow rate of about 1.1 L/min, and could operate continuously. Pure or distilled water was used as the electrolyte, and the combustion products were solely water vapor, ensuring zero pollutant emission. The device was equipped with an internal pressure monitoring system that automatically shut down when the pressure exceeded 0.5 MPa and resumed operation when the pressure dropped below the threshold, ensuring stable gas generation. For enhanced safety, the system included anti-flashback and explosion-proof protection units, and no gas was stored within the device. Even in case of malfunction, only a minimal amount of gas could undergo localized combustion, resulting in a very low safety risk.

**Figure 7:**
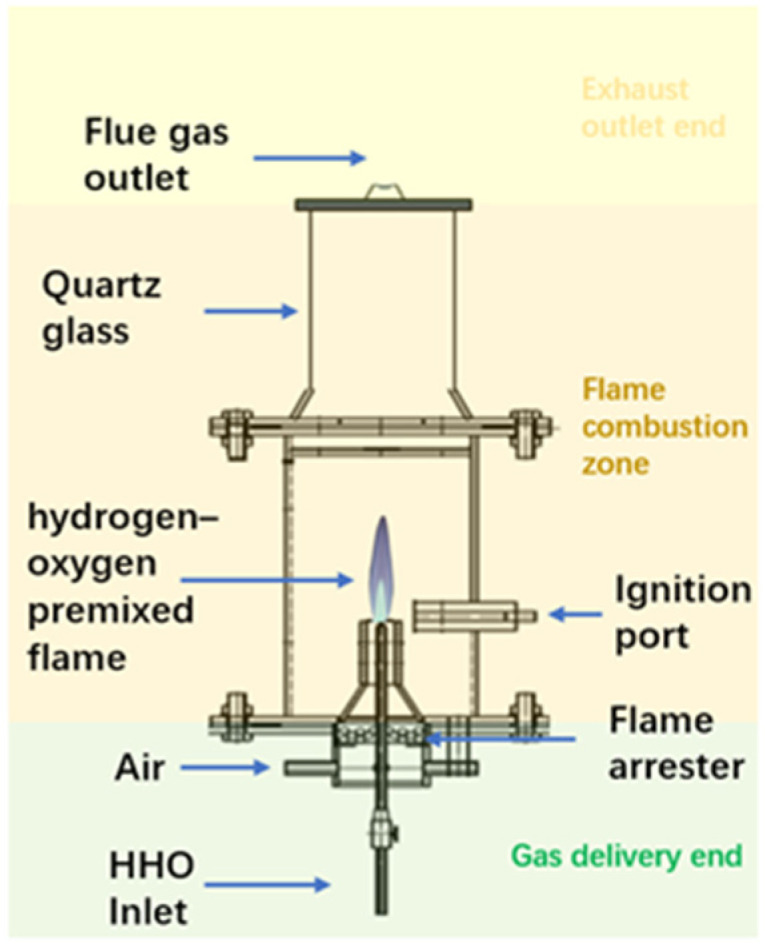
Burner structure.

The burner structure is shown in Figure 7. It consists of three main sections: the lower gas delivery section, the middle flame combustion zone, and the upper exhaust outlet section. The lower gas inlet adopts a T-shaped connector design, with the central tube used for introducing the hydrogen-oxygen mixed gas. The middle section serves as the flame combustion zone, composed of a quartz glass tube with an inner diameter of 50 mm and a height of 300 mm. Both ends are sealed with flanges and heat-resistant asbestos gaskets to ensure airtightness, and an electronic ignition device is installed near the burner nozzle. In the lower gas delivery section, the central passage supplies the hydrogen-oxygen mixed gas, while the outer passage delivers air. The addition of the air circuit ensures that an appropriate amount of air is introduced to dilute the hydrogen-oxygen mixture before ignition, thereby preventing explosive combustion.

### 3.2. Theoretical Basis and Model Construction of Quantitative OH-PLIF Measurement

(1) Relationship Between OH Fluorescence Signal and Radical Concentration

PLIF measurement is based on the selective excitation of specific electronic transitions. When a 283.62 nm laser irradiates the flame cross-section, OH radicals undergo an A^2^Σ^+^ ← X^2^Π transition, during which some molecules absorb energy and are excited to higher energy states. These excited-state molecules subsequently release energy through spontaneous emission or collisional quenching. Assuming the population of excited-state molecules remains in a steady state and that stimulated emission can be neglected, the number density of excited-state species satisfies a balance between excitation and quenching rates. The detected fluorescence signal intensity is proportional to the number of photons emitted per unit time by spontaneous emission from excited-state molecules. After accounting for the optical collection efficiency of the system, it can be expressed as:

(1) $$F=\frac{\eta \cdot \Omega \cdot V\cdot A_{21}B_{12}I_{v}f_{B}(T)}{4\pi (A_{21}+Q_{21})}\cdot N_{OH}$$

Here, *η* represents the total optical efficiency of the detection system, which includes the transmission efficiency of lenses, optical filters, and the detector response, and is primarily determined by the optical setup. Ω denotes the solid angle corresponding to the detector’s field of view, which depends on the lens aperture and the distance from the measurement region. *V* is the excitation volume, defined by the laser sheet thickness and the imaging field of view. *B*_12_ is the Einstein absorption coefficient [18] (m^3^·J^−1^·s^−2^), and *A*_21_ is the spontaneous emission rate (s^−1^). *I_ν_* represents the laser energy density per unit frequency (W·m^−2^·Hz^−1^), and *Q*_21_ is the quenching rate (s^−1^). *f*_B_(*T*) denotes the Boltzmann population fraction of the excited rotational level, which depends on the local flame temperature *T*. *Q*_21_ denotes the collisional quenching rate, which is strongly influenced by the concentrations of major gas species in the flame (such as N_2_, O_2_, and H_2_O) as well as the local temperature. *N_OH_* is the number density of OH radicals (m^−3^).

If all system-related parameters and temperature-dependent terms are grouped into a single proportional constant *C*, Equation (1) can be further simplified as:

(2) $$F=C\cdot \chi_{OH}$$

As shown in Equation (2), the OH-PLIF signal intensity exhibits an approximately linear relationship with the radical concentration; however, quantitative retrieval still depends on the accurate calibration of the proportional constant *C*. Since *C* encompasses multiple variables-including the optical system efficiency, the temperature-dependent Boltzmann population distribution, and the collisional quenching term-the derived concentration has only relative significance if independent calibration is not performed. To achieve quantitative analysis of fluorescence signals, absorption spectroscopy must be employed to obtain the actual OH radical concentration, while the absorption cross-section and population distribution are corrected using a two-line temperature inversion model. This approach enables the establishment of a calibration system applicable across different operating conditions.

(2) Absorption Spectroscopy and Two-Line Temperature Inversion Theory

To quantitatively calibrate the proportional constant *C* in Equation (2), absorption spectroscopy was introduced in this study to establish an independent concentration retrieval model. According to the Beer-Lambert law [20], when a monochromatic laser passes through a flame medium containing OH radicals, the transmitted laser intensity is related to the absorption cross-section and the OH number density as follows:

(3) $$I(\nu )=I_{0}(\nu )\cdot \exp[-\sigma (\nu ,T)\cdot N\cdot l]$$

In this equation, *I*_0_(*v*) represents the incident laser intensity (W·m^−2^), and *I*(*v*) denotes the transmitted laser intensity after passing through the absorbing medium (W·m^−2^). σ(*ν*,*T*) is the absorption cross-section (cm^2^), which characterizes the absorption capability of an individual particle per unit frequency interval and is a function of both the wavenumber ν and temperature *T*. *N* is the number density of the target radicals (cm^−3^), and *l* is the effective absorption path length (cm).

In practical combustion systems, the spatial distribution of target radicals along the laser propagation path is usually non-uniform. Directly using the geometric optical path length in place of the actual absorption length would introduce errors in the calculation of integrated absorbance. To accurately characterize the effective contribution of radicals within the absorption region, an effective absorption path length *l* is introduced. This parameter can be derived from the fluorescence gray-level distribution of OH-PLIF images at the same flame height, and is expressed as:

(4) $$l=\frac{1}{F_{0}}\int_{x_{1}}^{x_{2}}F(x)\mathrm{dx}$$

In this equation, *F*(*x*) represents the fluorescence gray-level distribution at a given flame height, *F*_0_ is the average gray level within the corresponding region, and *x*_1_ and *x*_2_ denote the boundary coordinates of the region with significant fluorescence intensity.

The two-line method determines the temperature by selecting two absorption lines within the same electronic transition band and calculating their intensity ratio *R* = *I*_1_/*I*_2_. The flame temperature can then be retrieved using the following expression:

(5) $$T=-\frac{\Delta E/k}{\ln(R\frac{S_{J^{\prime \prime }J^{\prime }1}}{S_{J^{\prime \prime }J^{\prime }2}})}$$

Here, Δ*E* = *E*_2_ − *E*_1_ represents the energy level difference between the two selected transitions, and *S_J_*_″*J*′_ denotes the corresponding line strength, which can be obtained from the HITRAN database [19].

(3) Integrated Absorbance and Temperature Inversion Model

To achieve quantitative retrieval of OH radical concentration, this study introduces a temperature-dependent correction model for spectral broadening under high-temperature flame conditions based on the Beer-Lambert law. Through a systematic derivation of the integrated absorbance model, the temperature-dependent term σ(*ν*,*T*) was explicitly expressed, and a coupling relationship between temperature correction and spectral broadening parameters was established. By analyzing the Doppler broadening effect caused by molecular thermal motion at elevated temperatures, a quantitative expression describing the variation in Doppler width with flame temperature was obtained, allowing the absorption coefficient α(*ν*) to dynamically respond to the actual temperature distribution within the flame. This theoretical model represents a mathematical extension of the previously introduced explicit temperature-correction formulation, providing a more precise theoretical foundation for subsequent temperature inversion and OH concentration calculations.

The absorption coefficient of a single spectral line follows a Gaussian broadening model, which can be expressed as:

(6) $$\alpha (\nu ,T)=S(T)exp[-(\frac{\nu -\nu_{0}}{\Delta \nu_{D}}{)}^{2}]$$

where α(*ν*,*T*) is the absorption coefficient per unit wavenumber (cm^−1^); *S*(*T*) is the line strength at temperature *T* (cm^−1^·(molecule·cm^−2^)^−1^); *ν*_0_ is the line center wavenumber (cm^−1^); Δ*ν_D_* is the Doppler width (cm^−1^), representing the spectral linewidth; and *v* is the wavenumber.

The Doppler broadening can be expressed as:

(7) $$\Delta \nu_{D}=\nu_{0}\sqrt{\frac{2kTln2}{mc^{2}}}$$

where *k* is the Boltzmann constant *k* = 1.38 × 10^−23^ J/K; m is the mass of an OH molecule; *T* is the flame temperature; and *v*_0_ is the line-center wavenumber taken from the HITRAN database [19], *v*_0_ = 31,000.06 cm^−1^.

The integrated absorbance *A* is calculated from the ratio of the transmitted intensity to the incident intensity:

(8) $$A=\ln\frac{I_{0}}{I}=\sigma (\nu ,T)\cdot l\cdot N_{OH}$$

By introducing the temperature-dependent absorption cross-section σ(*ν*,*T*), the integrated absorbance model can more accurately capture the quantitative relationship between OH radical absorption and temperature under high-temperature flame conditions, thereby achieving a dynamic response to the non-uniform temperature distribution within the flame.

## 4. Experimental Scheme and Operating Conditions

### 4.1. Experimental Conditions and Operating Procedure

To establish a quantitative relationship between OH fluorescence intensity and concentration, PLIF images must first be acquired under representative and stable conditions. In this study of hydrogen-oxygen premixed flames, multiple operating conditions were constructed by adjusting the hydrogen-oxygen flow rate to examine the response of OH production intensity to changes in fuel input, while identifying flame states with stable structure and high signal quality for subsequent calibration. The experimental system layout is shown in Figure 1; the apparatus has been described previously and is not repeated here. Below, the parameter settings and selection of the acquisition region are detailed in connection with the specified operating conditions.

The specific experimental operating conditions are listed in Table 3.

### 4.2. Laser System Configuration and Dye Wavelength Optimization

The laser system used in this experiment consisted of an Nd:YAG main laser and a Sirah dye laser, which were combined to generate excitation light at a specific wavelength to induce fluorescence from OH radicals. The procedure began by powering on the main laser and the dye laser. A laser energy meter probe was positioned at the Nd:YAG laser output to monitor the beam energy. The pulse delay parameter was set to 9 µs on the control panel, and the pump laser was activated. After the Nd:YAG laser completed preheating, the automatic crystal-angle adjustment function was initiated to optimize the internal crystal refraction. Once the adjustment was complete, the laser energy meter readings were observed to ensure a stable output energy of approximately 450 mJ, sufficient to drive the dye laser for subsequent excitation. The Nd:YAG laser produced a third-harmonic ultraviolet beam at 355 nm, which was directed into the Sirah dye laser after beam shaping to pump Rhodamine 590 dye, generating tunable laser radiation in the 283–284 nm range. To precisely excite the OH radical at the Q_1_(8) transition line, the internal resonator crystal angle of the dye laser was locked using Sirah Control v3.18 software to maintain a stable output wavelength throughout the experiment. Under the present operating conditions, the output energy was monitored by the laser energy probe, confirming that the single-pulse energy was maintained at approximately 20 mJ, with wavelength fluctuations controlled within ±0.01 nm, and the laser repetition rate fixed at 10 Hz.

To achieve precise matching of the excitation wavelength with the target spectral line, the Peakfinding module in the DaVis 10 software was used to scan the dye laser output wavelength and determine the optimal excitation wavelength. Under stable flame conditions, the output wavelength of the dye laser was scanned point by point over the range of 283.40–283.70 nm with a step size of 0.01 nm. At each wavelength point, the system automatically captured a single PLIF image and calculated its average gray-level intensity. Subsequently, the Peakfinding function generated a gray-level-wavelength response curve, as shown in Figure 3, representing the scanning results.

**Figure 8:**
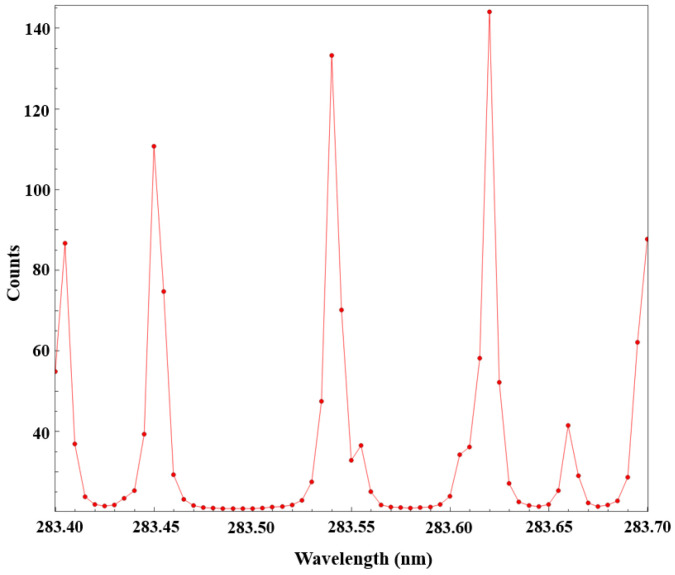
Peakfinding scan results.

As shown in Figure 8, the image gray-level intensity reaches its maximum at a wavelength of 283.62 nm, corresponding to the optimal excitation transition of the OH radical at the Q_1_(8) spectral line. This line within the OH (1,0) band exhibits the highest excitation transition probability, the longest fluorescence lifetime, and the lowest temperature sensitivity. After determining the optimal wavelength, the crystal angle of the dye laser was locked using the Sirah Control software to ensure wavelength stability throughout all subsequent experiments. Comparative analyses of multiple scanning results under different operating conditions confirmed that the gray-level values corresponding to this wavelength exhibited excellent consistency, demonstrating good repeatability and stability.

After configuring the laser system, precise synchronization between the laser pulse trigger and the image acquisition system was required to ensure accurate timing. The ICCD camera used in this study provides high temporal resolution and strong sensitivity in the ultraviolet range. The image acquisition system adopted the main laser pulse as the timing reference signal, with the gate width set to 1000 ns and the delay time to 700 ns. Experimental tests showed that, under this gate width, the image gray-level intensity approached saturation as the gate width increased, indicating that the fluorescence signal was fully captured while the noise level remained within an acceptable range.

To establish a physical mapping relationship between image gray levels and spatial positions within the flame, a field-of-view calibration was performed using a calibration ruler under natural light conditions. With the laser beam turned off, the ICCD exposure was adjusted, and the ruler was positioned at the burner outlet. The Scaling module in the DaVis 10 software was then used to calibrate the spatial scale. This procedure ensured that each gray-level point in the image corresponded accurately to its physical position in space, thereby providing spatial reference support for subsequent concentration retrieval.

### 4.3. Image Acquisition Procedure and Operating Condition Selection Strategy

After the laser system parameters were configured and the excitation wavelength was locked, a standardized image acquisition and processing procedure was established to ensure the accuracy of subsequent concentration retrieval and quantitative calibration. PLIF imaging was performed using an ICCD camera, and synchronization between the imaging system and the main laser pulse was achieved through TTL triggering, ensuring precise temporal alignment between laser excitation and camera exposure.

To determine the optimal acquisition parameters for OH-PLIF imaging, a scanning optimization of the ICCD camera gate width and exposure delay was conducted during preliminary experiments. The results indicated that a gate width of 1000 ns and a delay time of 700 ns allowed complete capture of the transient fluorescence emitted by excited OH radicals, while maintaining stable image gray levels and well-controlled background noise. The image intensifier gain was fixed at 75%, providing a suitable balance between signal amplification and image saturation.

During data acquisition, 200 consecutive instantaneous PLIF images were recorded for each operating condition. Image preprocessing was performed using the DaVis 10 software platform. The image processing procedure consisted of the following steps: (1) Background subtraction: A dark-field image was acquired under non-excitation conditions and used as a background template. Each PLIF frame was subtracted pixel by pixel to eliminate the influence of system background response and ambient stray light. (2) Laser energy normalization: Fluctuations in laser pulse energy can cause gray-level variations in the images. Therefore, the single-pulse laser energy was continuously monitored using an energy probe during acquisition, and the image gray levels were normalized according to the corresponding pulse energy. (3) Temporal averaging: To suppress local disturbances caused by flame fluctuations and enhance the signal-to-noise ratio, 200 consecutive PLIF images were averaged on a pixel-by-pixel basis according to the following expression:

(9) $$I_{avg}(x,y)=\frac{1}{N}\sum_{i=1}^{N}\left[\frac{I_{i}(x,y)}{E_{i}}\right]$$

where *I_i_*(*x*,*y*) represents the gray value at coordinate (*x*,*y*) in the *i*th image, *E_i_* is the corresponding laser pulse energy, and *N* denotes the total number of acquired images.

**Figure 9:**
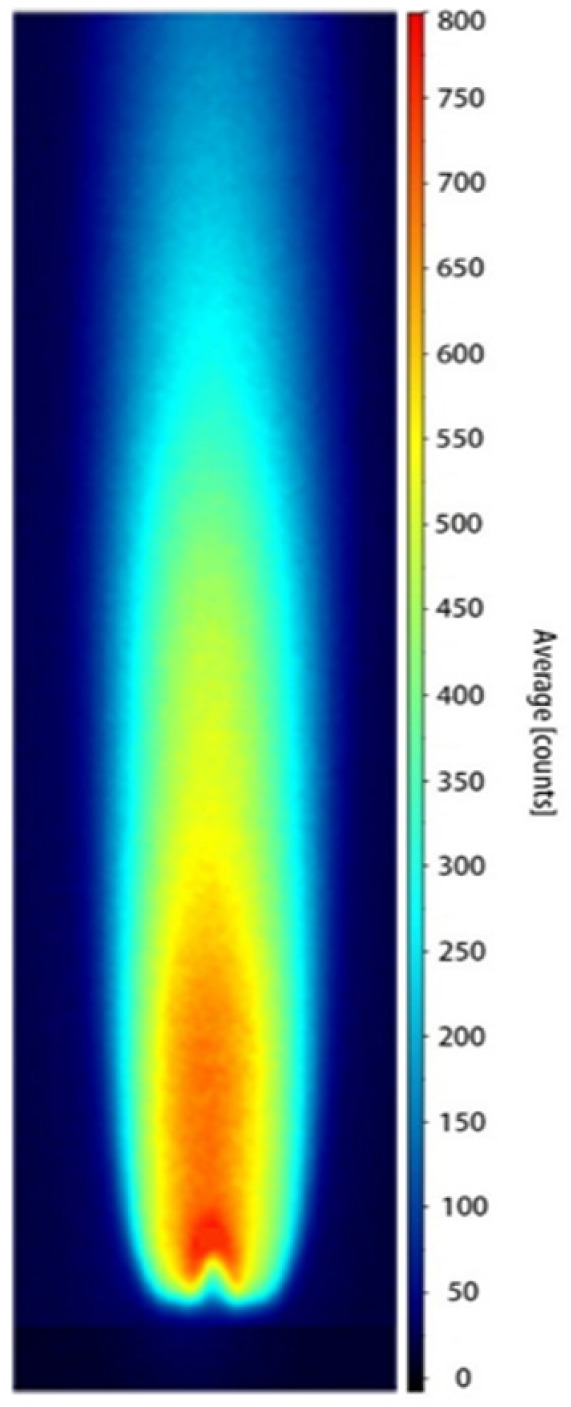
OH-PLIF image at a hydrogen-oxygen mixture flow rate of 0.8 L/min (the image was processed by background subtraction, laser energy normalization, and averaging over 200 laser shots, and the OH fluorescence was detected in the OH(A-X) emission band).

Figure 9 presents the processed PLIF image obtained at a hydrogen-oxygen mixture flow rate of 0.8 L/min, after background subtraction, energy normalization, and temporal averaging over 200 frames. As shown in the figure, the OH radicals are primarily concentrated along the central axis of the burner. The upper flame boundary exhibits a well-defined contour, and the fluorescence region maintains a stable morphology, providing a solid foundation for subsequent quantitative calibration.

## 5. Conclusions

Using a constructed hydroxyl flame experimental platform, combined with absorption spectroscopy and dual-line temperature inversion, this study investigated temperature correction and calibration for quantitative OH-PLIF measurements. By simultaneously acquiring laser absorption spectroscopy data and PLIF signals under various hydrogen-oxygen mixture flow conditions, a coupled correction relationship between integral absorption rate and flame temperature was established. Inversion results for OH radical concentration under multiple operating conditions were obtained, and the applicability of calibration constants was analyzed. Key conclusions are as follows:

(1) A quantitative calibration model for OH-PLIF based on absorption spectroscopy and dual-line temperature inversion was established. By introducing an explicit temperature-dependent absorption cross-section, the model achieved dynamic correction of the relationship between integrated absorbance and flame temperature.
(2) Experimental calibration was conducted under different hydrogen-oxygen mixture flow rates (0.4–1.2 L/min). The results show that the calibration constant *C* fluctuates by less than ±5% across all conditions, with the optimal constant determined as *C_opt_* = 0.01844. The average relative error under validation conditions was maintained within 4–6%, demonstrating good applicability and consistency of the calibration results.
(3) Compared with the uncorrected Matynia model, the overall error decreased from approximately 9.1% to 5.2% after applying the temperature correction, indicating that the inclusion of the temperature-dependent absorption cross-section significantly improves the quantitative calibration performance of OH–PLIF measurements.

## Figures and Tables

**Table 1:** Hydrogen-oxygen flame temperature inversion results.

Height (mm)	Hydrogen-Oxygen Mixture Flow Rate (L/min)	Fluorescence Intensity (283.455 nm)	Fluorescence Intensity (283.62 mm)	Temperature (K)
4	0.4	385	534	2048.9
	0.8	453	611	2269.3
	1.2	523	700	2340.3
10	0.4	354	492	2027.4
	0.8	424	585	2098.1
	1.2	476	651	2145.6
16	0.4	335	467	2013.6
	0.8	357	494	2058.7
	1.2	425	587	2077.5
22	0.4	312	441	1997.8
	0.8	346	478	2035.6
	1.2	350	481	2042.6

**Table 2:** Calculated calibration results of OH-PLIF under different hydrogen-oxygen mixture flow rates.

Hydrogen-Oxygen Flow Rate (L/min)	Incident Energy (mJ)	Transmitted Energy (mJ)	ln(*I*_0_/*I*)	OH Intensity at 4 mm	OH Number Density (mol·m^−3^)	Volume Fraction (ppm)	*C*
0.4	15.0	7.1	0.739	520	1.67 × 10^−1^	28,062	0.01853
0.8	15.0	6.8	0.793	580	1.77 × 10^−1^	33,320	0.017407
1.2	15.0	6.4	0.853	700	1.90 × 10^−1^	36,501	0.019178

**Table 3:** Experimental operating conditions.

Experimental Condition	Hydrogen-Oxygen Mixture Flow Rate (L/min)
1	0.2
2	0.4
3	0.6
4	0.8
5	1.0
6	1.2

## Data Availability

The original contributions presented in this study are included in the article. Further inquiries can be directed to the corresponding authors.
